# The legal feasibility of adopting a sugar-sweetened beverage tax in seven sub-Saharan African countries

**DOI:** 10.1080/16549716.2021.1884358

**Published:** 2021-04-20

**Authors:** Safura Abdool Karim, Agnes Erzse, Anne-Marie Thow, Hans Justus Amukugo, Charles Ruhara, Gemma Ahaibwe, Gershim Asiki, Mulenga M. Mukanu, Twalib Ngoma, Milka Wanjohi, Abel Karera, Karen Hofman

**Affiliations:** a SAMRC/Wits Centre for Health Economics and Decision Science - Priority Cost Effective Lessons for Systems Strengthening (PRICELESS SA), School of Public Health, University of the Witwatersrand, Johannesburg, South Africa; b Menzies Centre for Health Policy and Director of Academic Titles, School of Public Health, The University of Sydney, Sydney, Australia; c Community Health Department, School of Nursing, Faculty of Health Sciences, University of Namibia, Windhoek, Namibia; d School of Economics, University of Rwanda, Butare, Rwanda; e Economic Policy Research Centre (EPRC), Makerere University, Kampala, Uganda; f Health and Systems for Health Unit, African Population and Health Research Center, Nairobi, Kenya; g Health Policy and Management Unit, School of Public Health, University of Zambia, Lusaka, Zambia; h Oncology of the Ocean Road Cancer Institute (ORCI) and Oncology Department, Muhimbili University of Health and Allied Sciences, Dar Es Salaam, Tanzania; i Allied Health Department, School of Nursing, Faculty of Health Sciences, University of Namibia, Windhoek, Namibia

**Keywords:** Non-communicable diseases, NCD policy, fiscal policy, legal feasibility, sub-Saharan Africa

## Abstract

**Background**: A number of countries have adopted sugar-sweetened beverage taxes to prevent non-communicable diseases but there is variance in the structures and rates of the taxes. As interventions, sugar-sweetened beverage taxes could be cost-effective but must be compliant with existing legal and taxation systems.

**Objectives**: To assess the legal feasibility of introducing or strengthening taxation laws related to sugar-sweetened beverages, for prevention of non-communicable diseases in seven countries: Botswana, Kenya, Namibia, Rwanda, Tanzania, Uganda and Zambia.

**Methods**: We assessed the legal feasibility of adopting four types of sugar-sweetened beverage tax formulations in each of the seven countries, using the novel FELIP framework. We conducted a desk-based review of the legal system related to sugar-sweetened beverage taxation and assessed the barriers to, and facilitators and legal feasibility of, introducing each of the selected formulations by considering the existing laws, laws related to impacted sectors, legal infrastructure, and processes involved in adopting laws.

**Results**: Six countries had legal mandates to prevent non-communicable diseases and protect the health of citizens. As of 2019, all countries had excise tax legislation. Five countries levied excise taxes on all soft drinks, but most did not exclusively target sugar-sweetened beverages, and taxation rates were well below the World Health Organization’s recommended 20%. In Uganda and Kenya, agricultural or HIV-related levies offered alternative mechanisms to disincentivise consumption of sugar-sweetened beverages without the introduction of new taxes. Nutrition-labelling laws in all countries made it feasible to adopt taxes linked to the sugar content of beverages, but there were lacunas in existing infrastructure for more sophisticated taxation structures.

**Conclusion**: Sugar-sweetened beverage taxes are legally feasible in all seven countries Existing laws provide a means to implement taxes as a public health intervention.

## Background

The incidence of non-communicable diseases (NCDs) is increasing in the African region [1–3]. In particular, food-related risk factors, such as high-energy intake of sugar-sweetened beverages (SSBs), significantly contribute to obesity-related health conditions, including type 2 diabetes mellitus and heart disease [4,5]. Proactively addressing high rates of obesity in a cost-effective way will play a crucial role in reducing NCDs, which are projected to be the leading cause of death in the region by 2030, and will consequently reduce pressure on already strained healthcare systems [6]. Taxation and fiscal policies to improve public health are already employed, to varying degrees, in many countries, and can be effective mechanisms in NCD prevention [7,8].

Legal interventions play a key role in NCD prevention efforts at a population level by addressing risk factors, such as unhealthy diet, and alcohol and tobacco use [9–13]. This role is illustrated in the World Health Organization’s (WHO) best buys for NCD prevention, which require adoption through legal or regulatory instruments, such as increased taxation and reformulation of unhealthy products [11,14]. Although the impact of laws on public health is well-recognized [15,16], related research, on fiscal, regulatory or legislative interventions, has been subsumed under policy analysis. As a consequence, research focussed on efficacy and consideration of social, economic and political issues [16,17] can have a significant impact on the acceptability and feasibility of adopting a policy or intervention [18]. The TELOS rubric, which considers technical, economic, legal, operational and scheduling feasibility, has been used to assess health interventions [19,20]. Legal feasibility relates to the potential conflict with existing laws [19], while assessments of policy interventions include political acceptability, cultural and community acceptability, and trade-related legal feasibility [17,21]. Little consideration is taken of the legal implications of a policy intervention. Consequently, the regulatory/legislative nature of an intervention is omitted or not comprehensively considered in the analytical process.

SSB taxation is one example of a cost-effective legal intervention to control the rising burden of obesity in sub-Saharan Africa (SSA) [22]. Taxation is an avenue for governments to both reduce consumption of harmful products and generate revenue [23,24], which can be used for other prevention activities, such as health promotion or healthcare delivery [25]. Certain kinds of taxation structures can also prompt voluntary reformulation of harmful products [25]. The WHO recommends an *ad valorem* tax of 20% on SSBs to discourage consumption but does not prescribe the form that such a tax should take [22]. Different types of taxes that could be applied to SSBs are summarised in Table 1. In 2014, Mexico adopted a specific tax of 1 peso-per-litre (approx. USD 0.05) on SSBs [26]. In April 2018, both South Africa and the UK introduced SSB taxes [27]. The UK adopted a tiered tax, the amount of which depends on the sugar content (grams per 100 ml) of the beverage [27]. The tax is currently 18 and 24 pence per litre (approx. USD 0.23 and 0.31, respectively) for drinks with ≥5 and ≥8 grams of sugar per 100 ml, respectively [28]. In 2018, South Africa adopted a variable tax of 2.1 cents (approx. USD 0.0014) for every gram of added sugar above 4 g [29]. In December 2018, Colombia announced a value-added tax of 18% on carbonated beverages, pre-made teas and energy drinks [27]. Although it is too early to assess the public health impacts of some of these taxes, the tax in Mexico has been reported to have reduced consumption of SSBs [26]. The tax structures in South African provided an incentive for manufacturers to reformulate their products to reduce the amount of added sugar, leading to reduced sugar consumption [29].

**Table 1.** Types of taxes and their application to SSBs (adapted from Le Bodo et al. [37]

Type of tax	Explanation
Direct tax	Levied on consumer at point of sale, e.g. sales tax, value added tax
Indirect tax	Levied on goods at different points of the supply or value chain, e.g. an excise tax that is levied on a manufacturer
Specific tax	A fixed tax amount that is added to a product
*Ad Valorem* tax	Based on the value or price of the product, e.g. a percentage of the price
Volumetric tax	Based on the volume of produce, e.g. a tax levied per 100 ml or litre
Tiered tax	A differential tax rate based on, for example, the sugar or alcohol content of a product
Variable tax	A varying amount of tax based on, for example, sugar content; but may have a fixed rate.

Given the potential benefits to adopting an SSB tax in low- and middle-income countries (LMICs) and the variance in measures adopted, research is needed to understand what form an SSB tax could take in a particular country. While the efficacy and public health benefit of a taxation structure are important, it is equally important to choose a structure that complies with a country’s existing legal framework. Legal interventions cannot be assessed in the same way as ‘softer’ forms of policy. Laws are peremptory in nature, and mandatory – they impose obligations, require authority to be passed, and often impact a state’s obligations, particularly regarding human rights [12,14,30]. It is critical to ensure that there is no conflict with existing laws as this can render the intervention susceptible to challenges [30,31]. Despite the rigidities of legal considerations, in some instances, the only form that an intervention can take is a legal one. Since many NCD-related interventions conflict with the economic interests of multi-national corporations, they must fit within a state’s legal system, but be robust enough to withstand potential challenges [12,30,31]. Broadening the consideration of legal dimensions can lead to the adoption of more robust interventions, which can withstand potential legal challenges. Thus, it is critical to consider legal feasibility when determining whether such interventions should be adopted, but there is little guidance on how to do this [32].

Legal feasibility has often not been assessed when analyzing if and how taxes can be adopted in LMICs. At a local level, imposed taxes must be in accordance with the legal obligations of the state and within the ambit of the powers afforded to that level or arm of government [18,33], i.e. the appropriate taxation law must be used by the relevant government actors and/or institutions. In LMICs, it is also critical to ensure that the tax can be implemented effectively within the existing infrastructure [34]. There may be trade and investment law implications where fiscal measures favour local producers, and these should inform the taxation structure [35]. In summary, the implemented tax should be legally feasible and context-specific. There is then a need to consider, in the context of SSB taxation, which taxation structures could be implemented in a given country. This study sought to address this gap.

We assessed the legal feasibility of introducing or strengthening existing taxation laws related to SSBs, which could be leveraged for NCD prevention in seven SSA countries: Botswana, Kenya, Namibia, Rwanda, Tanzania, Uganda and Zambia. The aims were: a) to describe the landscape of existing laws related to SSB taxation, b) to analyse legal barriers and facilitators to the adoption of an SSB tax and c) to develop feasible formulations for SSB tax in each of the seven countries.

### Method

#### Study design

This study was conducted as part of a broader policy analysis, assessing the readiness of the seven SSA countries to adopt an SSB tax [36]. We undertook detailed analyses of the readiness of each country to adopt an SSB tax, utilizing the Kingdon Multiple Streams approach [36]. In this additional component of the study, we undertook to understand how existing laws could be barriers to, and facilitators of, the adoption of an SSB tax for the purpose of NCD prevention, drawing on the approach of Pomeranz et al. (2018) [33] in assessing the legal feasibility of SSB taxes.

### Designing legal feasibility methods for NCDs

NCD prevention efforts often have implications for sectors beyond health [11,31] and this should be taken into account in assessing legal feasibility. A detailed consideration of the legal feasibility of an intervention could inform how it could be formulated to: a) fit within the domestic legal system, b) meet government obligations, c) inform how other legislation and regulations can be harmonised with the intervention, and d) be robust enough to ameliorate the risk of legal challenge.

We conducted a literature review of methodologies used in legal feasibility studies on NCD prevention as described in Appendix 1. Our review included studies published from 2004 to February 2019, using Google Scholar, and the search terms ‘legal feasibility’ and ’non-communicable diseases’. We excluded studies that did not include legal feasibility within their methodology. Nine studies were selected for inclusion [10,17,32,33,37–41]. We identified commonalities, and strengths and gaps, in the approaches used, based on the context in which NCD prevention policies are adopted. We found that there was no standardised method for conducting legal feasibility assessments in NCD research or, more broadly, in public health research.

The methodologies we reviewed included some of the considerations listed above, but did not do so exhaustively and were not structured in a manner that allowed the methodology to be used in other legal systems, particularly in the LMIC context. The components of legal feasibility identified in the paper are available in Appendix 1. The lack of structure and uniformity in how a comprehensive legal feasibility assessment may be conducted is a key difficulty for assessing legal feasibility in the context of health, and NCD prevention, specifically. Although such an assessment can play a large role in shaping the form of an intervention [18], the legality of the intervention is more often than not viewed as a hurdle to be crossed or a binary consideration of whether interventions conflict with domestic law [17,32]. Given the potential value of a thorough legal analysis, there was a need to formulate a comprehensive and structured framework to assess the legal feasibility of interventions for NCD prevention in LMIC.

### The FELIP framework as conceptual model

**Figure 1.**
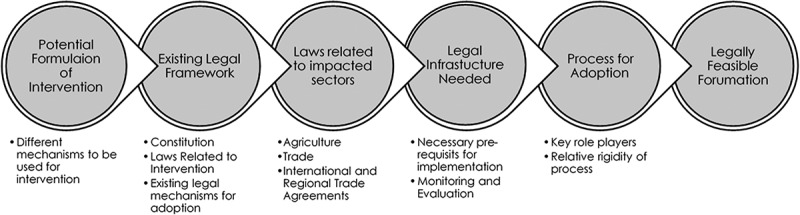
The FELIP framework for legal feasibility assessment

As none of the methodologies we identified could be utilised to address our aims, we developed a novel framework – the FELIP framework – which expands the utility of legal feasibility, allowing for a multiplicity of legal considerations to be analysed when assessing the legal feasibility of NCD-related interventions in LMICs. The specific components of the FELIP framework (Figure 1) are i) the potential **F**ormulations, ii) the **E**xisting legal system, iii) **L**aws related to impacted sectors, iv) legal **I**nfrastructure, and v) the **P**rocess. A detailed explanation of the meaning of each component and how it may be used to assess legal feasibility is outlined in Appendix 2. We used the FELIP framework to assess the feasibility of an SSB tax, taking the legal implications into account.

#### Application of FELIP framework to assess legal feasibility of SSB taxation

##### Selection of formulations of taxes

Following a review of recently implemented taxes, globally, we selected four formulations of an SSB tax adopted in other countries (Table 2). These taxes utilised different mechanisms and provided a good basis from which to assess the feasibility of a broad range of SSB taxation mechanisms.

**Table 2.** Taxation formulations adopted by Mexico, UK, South Africa and Columbia

Country	Tax Formulation
Mexico	Specific volumetric tax
UK	Tiered tax with differential rates based on sugar content
South Africa	Variable tax based on sugar content
Columbia	Removal of a VAT exemption on SSBs – an effective direct tax of 19%

We analysed the following tax formulations: 1) volumetric tax rates, 2) variable taxation based on sugar content, 3) tiered taxation based on sugar content, and 4) value-added tax (VAT) exemptions. The first and second tax formulations were selected because they are commonly applied to SSBs and sugar in Africa and globally. The third, VAT exemption, was included because it may be a mechanism to enable easier access to healthy food. In addition, removing VAT exemptions of SSBs and related products is important to ensure policy coherence.

During the review, we found that a few countries, such as Tanzania and Kenya, had introduced sugar levies, which were not included in our selected formulations. We included these sugar levies in our analysis because they are not generally implemented in high-income countries, but are of particular relevance in sugar-producing countries.

##### Existing legal framework and laws related to impacted sectors

We reviewed the legal documents related to SSB taxation in each country; these included domestic constitutions, value-added tax legislation, and excise tax legislation. To establish the position of each country with regard to sugar levies, we evaluated budget documents to assess whether there was any income generated from sugar levies, and utilised this information to locate the relevant legislation. We used African LII, and country-specific legal information institute websites, such as UgandaLII and KenyaLII, to identify laws, regulations and policies by searching the country name and the key phrases, ’sugar law’, ‘sugar levy’, ’duty on sugar’ or ’sugar tax’. We excluded documents pertaining to import duties on sugar.

The complicated nature of trade agreements, particularly bilateral trade agreements, means that the trade environments differ by country. For this reason, and given the inclusion of seven countries, our review of regional legal documents was limited to the health-related treaties under the African Union, the Southern African Development Community (SADC) Protocol on Trade, and the Treaty for the Establishment of the East African Community (EAC). These documents were obtained by searching each institution’s websites for health-related treaties and manually reviewing the treaties to extract relevant provisions.

Data collection took place in March 2019. The seven countries’ constitutions were accessed through the Constitute Project [42], which maintains a database of country constitutions. The constitutions were reviewed to establish whether they contained self-standing rights to food and/or health, whether they included any provisions to protect health, and whether they linked food to health in any way. The excise tax, levy, VAT and nutrition labelling laws and regulations for the seven countries were accessed from country-specific government websites or legal information institute databases. The information was supplemented with tax guides from the relevant country revenue authorities, budget speeches, and news articles on taxation of local sugar industries. Some of the laws reviewed contained provisions on import duties on sugar; we excluded these provisions from the review. For the regional trade-related documents, we used treaty-specific websites to establish whether the countries were signatories to the treaty and downloaded copies of health-related treaties.

##### Infrastructure

To determine whether the necessary infrastructure was in place to support SSB taxation in each country, we established whether there was a mechanism to report the sugar-content of beverages, using a nutrient information label. We used the LII database to search for nutrition labelling laws and/or provisions for the monitoring and testing of the nutritional content (specifically sugar) of different products.

##### Processes

To establish the process for lawmaking in each country, we relied on the publicly available process outlined by national lawmaking bodies. We searched each country’s parliamentary websites for summaries of the law making-process and identified the relevant policymakers involved in the process of passing tax laws in each country.

### Analysis

The data from all relevant identified documents were extracted (category of law, provisions related to SSBs, taxes or sugar, contents and scope of the provision including tax structure and rates, and policymakers involved) and analysed, using the FELIP framework components outlined in detail in Supplementary section 2. The summary of the data extracted is outlined in Table 3.

**Table 3.** Summary of key legal provisions related to SSB taxation in seven sub-Sarahan countries

		Botswana	Namibia	Kenya	Uganda	Rwanda	Tanzania	Zambia
Existing Legal Framework	**Constitution**	Limitations clause for public health purposes	Right: food (acceptable level of nutrition to improve public health)	Right: Food (free from hunger) and health	Limitations clause for public health purposes	Right: health (facilitate activities aimed at good health)	Limitations clause for public health purposes	Limitations clause for public health purposes
	**Tax Laws** (SSBs/sugar taxed)	Excise tax (not SSBs) VAT exemption (sugar)	Excise tax (not SSBs) VAT exemption (sugar)	Excise tax (SSBs): differential tax rates for certain beverages VAT exemption (sugar)	Excise tax (SSBs): differential tax rates for beverages Levy for HIV fund: all non-alcoholic drinks	Excise tax (SSBs): differential tax rates for beverages	Excise tax (SSBs) VAT exemption (sugar cane)	Excise tax (SSBs): differential tax rates for certain beverages*
	**Taxation Rates**	VAT: 12%	VAT: 15%	Excise tax on SSBs: 10 Shs/litre VAT: 16% Sugar Levy: 4%	Excise tax on SSBs: 13% or UGX 240/litre (the higher of) VAT: 18% Sugar levy: 1% HIV Fund Levy: 2%	Excise tax on SSBS: 39% VAT: 18%	Excise tax on SSBs: 54 tshs/litre VAT: 18% Sugar levy: unclear	Excise tax on SSBs: 30 ngwee/litre* VAT: 16%
Laws related to impacted sectors	**Agriculture**			Sugar development levy: to assist producers	Sugar levy: for revenue generation		Sugar development levy: to assist producers	
	**Trade and investment**	SADC AU	SADC AU	EAC AU	EAC AU	EAC AU	EAC AU	SADC AU [Domestic] Tax allowances for SSB manufacturer removed
Legal infra-structure	**Nutrient Label Laws**	Regulation in place	Regulation in place	Regulation in place	Regulation in place	Regulation in place	Regulation in place	Regulation in place
Processes	**Key Policy Makers**	President	Attorney-General	Finance Committee and Minister of Finance	Minister of Finance		President and/or Minister of Finance	Any minister
	**Parliamen-tary vote required**	Yes	Yes	Yes	Yes	Yes	Yes	Yes

*Law not in force at time study was conducted.

We then coded the provisions of the laws according to which FELIP component they applied to (**e**xisting law, **l**aws related to impacted sector, legal **i**nfrastructure or **p**rocess). Each provision was coded as supportive, conflicting, compatible or not applicable to each of the selected SSB taxation formulations.

A researcher from each country validated the extraction template to ensure accuracy and utilised consultations with key informants to validate the completeness and accuracy of the documents analysed [36]. The data for each component of the FELP framework were then synthesised utilising the framework to identify barriers (in the form of conflicts with existing laws) and facilitators (legal mandates that required action and existing laws that could be adapted) to assess the feasibility of each of the tax formulations in the seven countries. A independent trained lawyer, not involved in the study, reviewed the coding and synthesis to ensure the accuracy and objectivity of the analysis.

## Results

Many of the countries had existing taxation laws in place but, surprisingly, many already taxed SSBs and there was existing infrastructure that could be used for the adoption of an SSB tax. Table 3 outlines the key legal provisions related to the following components of the FELIP framework: ii) the Existing legal system, iii) Laws related to impacted sectors, iv) legal Infrastructure, and v) the Process.

### Existing legal frameworks for taxation

#### Enabling provisions in domestic constitutions

Broadly speaking, the constitutional provisions reviewed can be categorised as ‘limitations clauses’ or ‘self-standing rights’. Limitations clauses do not confer an entitlement or obligation but may allow the infringement of rights if they serve a public health purpose. Self-standing rights give citizens an entitlement to exercise their rights and place obligations on government to take steps to fulfil those rights. The existence of a limitations clause or a self-standing right to health or nutritious food will facilitate and support the adoption of measures such as an SSB tax.

Of the seven countries reviewed, Kenya, Namibia and Uganda had self-standing rights related to food or health, and Botswana, Tanzania and Zambia allowed other rights to be limited for public health purposes. Rwanda did not have any supportive clauses but there were no clauses that would prevent the adoption of an SSB tax. All of the constitutions permitted government to levy taxes. Thus, an SSB tax was permitted as a taxation measure in all seven countries but there was support for it as a public health measure in six.

#### Regional agreements requiring action on NCDs

All seven countries are members of the African Union. Botswana, Namibia and Zambia are members of SADC; Kenya, Rwanda, Uganda and Tanzania are members of the EAC. The African Charter on Human and Peoples’ Rights, 1981, provides that ‘every individual shall have the right to enjoy the best attainable state of physical and mental health’ and that parties to the treaty shall take measures to protect the health of their populations. The Protocol on Health in the SADC has specific provisions related to NCDs. Article 13 requires that state parties co-operate and assist one another to ”adopt appropriate strategies for the prevention and control of [NCDs]”. The EAC Treaty requires that members undertake to ‘take joint action towards the prevention and control of … non-communicable diseases … ’ as well as ‘promote the development of good nutritional standards’. At a regional level, there is a clear mandate to take action on the prevention and management of NCDs, which will buttress the validity of an SSB taxation if such a measure is challenged by other members in the SADC or EAC.

#### Existing taxation policies related to non-alcoholic, soft drinks and SSBs

Excise tax is commonly applied to non-alcoholic and soft drinks, and this tax therefore also applies to SSBs. Four countries (Kenya, Tanzania, Uganda and Rwanda) had excise taxes on SSBs in place (Table 3) – an indirect tax levied on the manufacturer. The two countries (Botswana and Namibia) that did not impose excise taxes on SSBs had excise tax legislation in place. At the time of this study, the Zambian government had announced its intention to tax non-alcoholic drinks utilising an excise tax, specifically for NCD prevention, in 2019. The bill to introduce this, and other changes, was on its third reading in the Zambian Parliamentat the time of this study.

In the four countries that imposed excise taxes, and in Zambia, the formulation varied substantially. Kenya, Tanzania and Zambia had volumetric taxes in place, while Uganda and Rwanda had valoric taxes. The laws in Kenya and Tanzania had separate categories for sweetened and unsweetened beverages in the tariff codes. None of the existing laws had a tax based on sugar content, but Kenya, Rwanda and Uganda taxed certain beverages at different rates. Although there were differential tax rates, most legislation (except that of Zambia) did not have different rates for SSBs and unsweetened beverages. Consequently, the excise tax framework would be a potential route for the adoption of health-related taxes.

Although some of the countries had VAT exemptions for sugar, none had exemptions for SSBs. Since many of the countries already impose VAT on SSBs, removal of a VAT exemption was unnecessary.

### Laws in related sectors

Since SSB taxes can impact sectors such as agriculture and health, we analysed how existing fiscal policies related to these sectors could be leveraged or addressed to disincentivise consumption of SSBs and/or sugar. This consideration yielded a number of pathways to implement fiscal policies on SSBs not often considered in high-income settings.

#### Agriculture

The three east African countries (Kenya, Uganda and Tanzania) impose levies on sugar producers, generating either revenue for the state (Uganda) or funds to support the activities of sugar producers (Tanzania and Kenya). Specifically, Tanzanian’s sugar levy is used to finance the Sugar Development Fund, the key activities of which include sugar marketing and promotion, provision of financial assistance to cane development and sugar plants, and training. We were unable to locate an authoritative source for the rate of the levy. Kenya had a 4% sugar development levy in place until 2016, when it was removed [43]. This levy was intended to fund cane development and factory rehabilitation; 2019 news reports indicated that the industry had been lobbying for the levy to be reintroduced [43,44].

#### Health

At the time this study was conducted, the only health-related SSB tax was the announced Zambian excise tax. Uganda had one health-related fiscal measure: an HIV/AIDS-related act established a levy on beers, spirits, *waragi* (a form of domestically distilled spirits), soft drinks and bottled water, the proceeds of which are earmarked for the HIV/AIDS trust fund. This provides an alternative to the introduction of an excise tax. A levy in this form allows funds to be ear-marked for health purposes, and the law could be amended to add NCDs to the existing HIV/AIDS infrastructure and address NCDs as a multi-sectoral issue. In addition, it is not subject to the same rigid parliamentary processes as tax legislation.

### Legal infrastructure to support SSB taxation

Food labelling is essential to monitor and assess the sugar content of SSBs, and provides the infrastructure necessary for SSB taxation. All seven countries had regulations or legislation providing for food labelling, even where an SSB tax had not been implemented (see Table 3). However, if a tax based on the sugar content of a beverage is implemented, there is still a need for local revenue authorities to put measures in place to facilitate the assessment and collection of these taxes.

### Processes for taxation adoption

Overall, the processes for passing legislation are similar across the different countries. Typically, a bill is drafted by a relevant policymaker (a committee or a member of cabinet). It is then considered and passed by Parliament before being sent to the President for signature. The consideration by Parliament may take the form of a number of readings or may comprise a brief debate and vote. In most countries, the President has the power to veto the bill by choosing not to sign it and returning it to Parliament for reconsideration. In Namibia, there is an additional policymaker involved as draft bills first go through the attorney-general to ensure that they align with the country’s constitution. Most countries have an expedited parliamentary process for tax or money bills. As outlined in Table 1, some countries require the specific involvement of key policymakers, such as the Minister of Finance (Kenya, Uganda) or the President (Botswana and Tanzania).

## Discussion

In this study, we sought to use existing laws to inform the design of a context-specific tax on SSBs and to test the feasibility of different tax designs in SSA. Despite differences across the seven countries, we found that utilising an existing excise tax infrastructure is a feasible route to follow, should countries opt to implement the WHO recommendation of an taxation rate of 20% targeting SSBs.

Table 4 outlines the legally feasible mechanisms that could be used in each of the seven countries. All have excise taxes in place and a majority levy an excise tax on SSBs as part of their taxation of non-alcoholic and soft drinks.

**Table 4.** Potentially feasible formulations of SSB taxes in seven SSA countries

Tax formulation	Botswana	Namibia	Kenya	Uganda	Rwanda	Tanzania	Zambia
**Specific tax**	Possible to use excise tax	Possible to use excise tax	Tax exists but requires differential rates for SSBs	Tax exists but requires differential rates for SSBs	Tax exists but requires differential rates for SSBs	Tax exists but requires differential rates for SSBs	Tax exists but requires differential rates for SSBs
**Tiered tax**	Preliminary infrastructure in place Implementation may be difficult through indirect taxes						
**VAT exemption**	No VAT on SSBs Consider harmonisation by removing exemptions on sugar where applicable						
**Existing levies**			Possible to reinstate previous sugar levy to assist sugar industy to diversity	Possible to repurpose existing sugar or HIV levies which are already earmarked		Possible to repurpose existing sugar levy	

Six of the seven countries included in the study have a mandate to improve their population’s health. Although not all countries have an excise tax on SSBs, all seven have excise tax legislation, which can be utilised to introduce an SSB tax. The advantage of pursuing an existing taxation mechanism in many of these countries is that there is an expedited parliamentary process which allows for quicker, more streamlined implementation. However, passing fiscal legislation often also requires the buy-in of specific policymakers, such as treasuries, Ministers of Finance or Presidents. Where there is a lack of buy-in from these key policymakers, it may be preferable to consider alternative avenues for reducing SSB consumption. The role of these policymakers requires examination as part of a political feasibility analysis. Overall, an excise tax, either as a flat rate or based on sugar content, is a legally feasible route to curb SSB consumption.

None of the seven countries reviewed have a VAT exemption or zero-rating for SSBs, so this formulation of an SSB tax is not feasible. However, the existence of other mechanisms, such as sugar levies, presents an alternative to taxation-related interventions. These alternative mechanisms might be of use where there is a lack of buy-in from policymakers. This is illustrated by Uganda’s HIV levy. In its current structure, the levy targets a variety of drinks, some of which are not unhealthy, such as bottled water, and imposes a rate of 2%, which is lower than the WHO recommendation of 20%. Consequently, if utilised to impose an SSB tax for NCD prevention, we would recommend that the levy target only unhealthy products and that the rate be raised.

Although all the countries could utilise an excise tax mechanism, there are also potential opportunities to pursue a different tax structure that might allow for ear-marking of … … or utilise a more flexible legislative process. In addition, we found that, in certain countries (such as Namibia and Rwanda), the adoption of an SSB tax would assist governments in meeting their constitutional obligations.

The findings from this study demonstrated that it is necessary to consider the broad legal framework in place in each country when determining which tax formulation to adopt, as there are formulations that may be wholly excluded. For example, removing a VAT exemption would not be an effective intervention as none of the countries had VAT exemptions of SSBs. However, many countries had VAT exemptions on sugar and governments could consider removing these exemptions to disincentivise the consumption of sugar more broadly.

In addition, this comprehensive approach to legal feasibility exposed potential mechanisms that could be married with the adoption of a tax to appease opponents from the agricultural or trade sections, such as an agricultural levy. Although agriculture levies present an opportunity for SSB taxation, there are inherent tensions between measures, such as sugar development levies and interventions that seek to reduce consumption of sugar. As in the case of VAT exemptions of sugar, it will be necessary for governments to consider removing these incentives and supports for sugar production or redirect them towards activities to diversify the sugar industry.

The most surprising finding from this analysis was that five countries already levied an excise tax on carbonated beverages, either as a form of revenue generation or, in the case of Zambia, as a proposed public health measure. These existing taxes present an opportunity to leverage off of an existing income stream for governments and to advocate for the adoption of differential rates that both meet public health objectives and provide additional revenue to resource-constrained governments.

The structure and influence of regional trade and taxation agreements is an area that requires further research. Arrangements between SADC members create different – and sometimes competing – priorities for countries, which may help or hinder the adoption of an SSB tax. At present, the SADC seeks to harmonize tax measures across the region but also seeks to create coordinated tax incentives to support trade and foreign investment [45]. The impact of South Africa – and now Zambia – adopting taxes on SSBs may open an opportunity for SSB taxes to be harmonized and implemented across the trade block. However, the tax exemptions and investment allowances are prioritized by the SADC to lighten the tax burden on business, irrespective of the business’ impact on health [45]. There are inherent tensions between these SADC goals and measures, such as those taken by Zambia, to lessen the incentives for the SSB industry. In addition, there may be broader trade concerns, including equal treatment and non-discrimination concerns, which may leave measures susceptible to challenges that we did not investigate. At the same time, the regional commitments to prevent and control NCD place obligations on governments to be responsive in addressing NCDs. As we have shown, trade laws and the broader legal system of a country may serve as barriers to, or facilitators of, the adoption of an SSB tax, depending on the context and structure of the tax.

### Limitations

This was a desk-based study and is, consequently, subject to several limitations. We relied on data sources that provided open access to laws, such as country-based Legal Information Institutes. These platforms are not always up-to-date and, as a result, some of the laws we reviewed may not reflect recent amendments. The data collection was conducted in March 2019 and the analysis was based on the status of the laws as at that date. We attempted to compensate for this by validating the accuracy of documents with local policymakers but, in some instances, we were unable to obtain copies of authoritative legal sources for certain provisions as noted in the results. A further limitation is that there was no standardised method available for conducting legal feasibility analysis for public health interventions. We addressed this by developing a framework that could be used to standardise the components of legal feasibility analysis for public health interventions. The analysis was validated by an external lawyer not involved in the study to address potential for bias or inconsistency in the legal analysis. We excluded most of the trade-related implications of an SSB tax from this study due to our scope, and this is an area that requires further research.

## Conclusion

An SSB tax aimed at preventing obesity and nutrition-related NCDs is legally feasible in the seven SSA countries; there are no legal barriers to the adoption of such taxes. Countries have either constitutional or regional mandates to take action to prevent NCDs, which must be discharged. The adoption of an SSB tax provides an opportunity to meet these obligations. The findings from this study can be used to improve the design of existing taxes or offer new ways for governments to disincentivise the consumption of SSBs. In this sense, we also reinforced the significant role that law, and taxes, may play in NCD prevention, and the need to comprehensively consider local laws before implementing an intervention.

## Paper context

Taxation of sugary beverages is a recommended intervention to prevent non-communicable diseases. When adopted, sugar-sweetened beverages taxes vary and there is a need to integrate these taxes into existing domestic legal systems. We assessed the legal feasibility of adopting sugar-sweetened beverage taxation in seven sub-Saharan African countries. We found that taxation of sugary beverages is legally feasible in all the countries and that existing excise laws provide a potential pathway to adoption.

## Appendix 1: Literature review of Legal Feasibility Studies

### Selection of studies for literature review

We utilised Google Scholar for the literature review as many trusted databases such as PubMed do not include studies or papers from legal journals. The search on GoogleScholar generated 18 600 results. However, a review of the titles showed increasingly irrelevant results after the first 35 results. To make the review comprehensive, we reviewed the abstracts of the first 200 results. We included 21 papers that mentioned legal feasibility in the abstract. We then reviewed the methodologies of these papers and excluded papers that did not include legal feasibility in their methodology. This last screening led to the selected the 9 papers.

### Overview of methodologies to assess legal feasibility

There were five different methodologies used, the most common being that described by Snowdon et al. [1] (the Snowdon Methodology), which is a broader policy analysis methodology. Although this was the most common methodology, legal feasibility plays a small role in the Snowdon methodology. The methodologies of Wilde et al. [2] and Pomeranz et al. [3] (the Wilde and Pomeranz methodologies, respectively) did not assess legal feasibility exclusively, but as a component of broader feasibility. Bodo’s study only considered legal feasibility.[4] Graff et al. [5] (the Graff methodology) did not undertake a feasibility assessment but provided a potential framework for future legal feasibility studies.

**Table:** Components of Review of Legal Feasibility Methodologies

	First author				
Component	Graff	Snowdon	Wilde	Pomeranz	Bodo
Country of legal system	USA	Pacific islands (and others)	USA	USA	Canada
Considers only legal feasibility	**X**				**X**
Authority	**X**		**X**	**X**	**X**
Pre-emption	**X**		**X**	**X**	
Infringement on Rights	**X**		**X**		
Impact on government liability	**X**				
Trade-related Concerns		**X**			
Case Law			**X**		
Existing laws related to intervention			**X**	**X**	**X**
Lessons from previous similar interventions			**X**		**X**
Considers how the law may facilitate adoption of intervention			**X**		**X**

### Key components of existing legal feasibility methodologies and shortcomings

#### Overview

We found that, due to most of the studies approaching legal feasibility from the perspective of the US legal system, the majority of studies limited the assessment of legal issues to whether government had the authority to adopt the intervention and whether it conflicted with existing laws, particularly trade-related implications.[1,5–8] Many studies, drawing on Snowdon et al, had trade law implications as the sole law-related consideration.[1,6–8] A few, more detailed legal feasibility assessments considered which existing laws and how these could be amended to include interventions.[2–5] In a limited sense, these assessments used the law to inform how the intervention could be structured but the factors considered were largely specific to Canada and, particularly, the United States of America. These methodologies emphasised the protection of civil and political rights as well as concerns around pre-emption. [2–5]. Consequently, issues such as consideration of socio-economic rights and the role of policies and laws related to food production, foreign investment and trade, which arise more in a LMIC context, [9] were overlooked.

#### Authority

The first leg of the Graff methodology is whether the relevant branch of government (often the legislature) has the necessary authority to adopt an intervention and this should arguably be a primary consideration to assess the feasibility of any legal intervention.[10] This is similarly the initial consideration in Bodo and Wilde’s methodology.[2,4] If the legislature (or other relevant branch of government) lacks the authority to adopt the intervention, there is no need to consider anything further as often any actions taken outside this authority will be invalid.^2^ Wilde et al do consider whether the intervention falls within the broader powers of the relevant arm of government as a potential facilitator. [2] However, the framing of this aspect is largely concerned with whether this intervention falls within powers of the arm of government seek to adopt the intervention.

Though authority is central, it is not the only relevant consideration in this regard. For example, where a country’s constitution contains socio-economic rights, it would be equally important to assess whether legislature was mandated or obligated to pass an intervention. Over 135 domestic constitutions, including LMIC like El Salvador, Kenya, Peru and Togo now recognise a right to health and some jurisdictions have entrenched this as a justiciable obligation.[11] Many countries have also signed international treaties on socioeconomic rights such as the International Covenant on Economic, Social and Cultural Rights [12]. Thus, governments may in fact be compelled to take action to improve or protect public health depending on how these rights operate in each country. This question of authority, in a way, links to the third leg of the Graff methodology which requires that the intervention not infringe on any constitutionally protected liberties.[5] This requirement is of value in contexts where there are no limitations clauses or provisions protecting socio-economic rights. However, where a constitution contains socio-economic rights and / or internal limitation clauses, there may be tensions between these rights and other civil and political rights which do not automatically render the intervention indefensible because the right to health warrants equal protection to civil entitlements.[13,14] Some constitutions contain caveats which allow civil liberties to be infringed for public health purposes, thus facilitating the adoption of health-related interventions.^3^

#### Pre-emption

Though pre-emption is closely linked with authority, it arises almost exclusively in strongly federalist systems where action by one arm of government might invalidate action by another and is concerned with whether the intervention might conflict with existing laws or have some other detrimental effect.[15,16] Issues of pre-emption broadly relate to a branch of government’s power to take certain actions.[15] Bodo’s consideration of multi-level government powers uses this to inform what the different formulations of an SSB tax might look like depending on which arm of government adopts the tax.[4] Wilde et al adopt a similar approach in suggesting which level of the legislature can pass specific laws and using this to outline the varying feasibility across the different levels.[2] This approach is preferable as it creates a developmental aspect which informs how the intervention might be have to be changed depending on the branch of government adopting it rather than a binary assessment of whether it is permissible. Going beyond pre-emption in this fashion expands this to a broader consideration of authority.

#### Options for adoption

Bodo, Pomeranz et al and Wilde et al outline the different formulations one could use to adopt the health intervention but their approach to determine which formulations should be considered differs. Pomeranz et al undertakes a systematic review of SSB and junk food taxation structures under both domestic and international law.[3] The formulations the Pomeranz methodology considers are triaged to focus on taxation structures for public health and existing taxation structures. Bodo considers all potential taxation structures before determining which would be appropriate for SSB taxation with more limited reference to scientific literature.[4] The Wilde methodology begins with a comprehensive review that is reduced to four discrete policy interventions for limiting consumption of processed meat which are not exclusively legal in nature.[2] What is of relevance from these differing approaches is the indication that there may be a variety of interventions that should be considered. When utilised in decision-making by policymakers, there is a need for flexibility in legal feasibility which would allow the assessment of a number of different interventions which may differ in their form and mechanisms for adoption. In some instances, legal feasibility may be used to decide between three or four taxation structures while in others, it may inform a decision between improved labelling laws and reformulation.

#### Existing laws

Bodo, Wilde et al and Pomeranz et al all give consideration to the existing laws which apply to the intervention though the lens with which this is done differs. Bodo utilises the existing laws to inform the formulation of the intervention of the specific legal context.[4] This is an important dimension of legal feasibility which allows a general recommendation to be translated into a specific action for a country to adopt. However, the ambit of existing laws considered by Bodo limits the value of this process solely to tax laws irrespective of the other sectors which may be impacted by the intervention. Pomeranz et al and Wilde et al look to precedent or existing laws where a similar policy has been adopted, though Wilde et al place greater emphasis on this consideration.[2,3] This is useful as it demonstrates how a similar measure was adopted in the same legal context and can also provide an entry point to the intervention through amending existing legislation.

However, NCD prevention efforts often have implications for sectors beyond health [17,18] and this should be taken into account in assessing legal feasibility. In particular, one should consider whether there are any laws which might serve to undermine the effectiveness of the intervention. This concerns laws which in substance and effect have an opposite effect to the intervention, such as a sugar subsidy undermining an SSB tax. This may not inform the formulation of the intervention but can inform broader recommendations to harmonise conflicting laws.

However, NCD prevention efforts often have implications for sectors beyond health [17,18] and this should be taken into account in assessing legal feasibility. In particular, one should consider whether there are any laws which might serve to undermine the effectiveness of the intervention. This concerns laws which in substance and effect have an opposite effect to the intervention, such as a sugar subsidy undermining an SSB tax. This may not inform the formulation of the intervention but can inform broader recommendations to harmonise conflicting laws.

#### Trade-related implications

Though not much detail was provided to indicate how the trade-related legal issues were assessed, they remain a critical factor in determining whether an NCD prevention intervention will be viable.[19,20] Historically, international trade and investment law has been used by the tobacco industry to prevent the implementation of tobacco control measures through institutions like the World Trade Organisation.[18,21–23] Though these cases largely upheld the interventions, there is less guidance and clarity on the position for food-related interventions. More recently, trade concerns regarding food labelling measures adopted by Chile, Ecuador, Indonesia, Peru and Thailand have been filed in the WTO.[24] In addition, regional trade agreements may impose further restrictions on the adoption of measures that may create barriers to trade.[25] It is then necessary to consider whether the intervention may have implications under existing trade agreements.

### Appendix 2: Detailed explanation of the FELIP Framework

Legal considerations can also inform how an intervention should be formulated. Where constitutions, policies or other legal infrastructure provide the government with a mandate to adopt health interventions, the legal system can be supportive.[26] In addition, considering existing legal frameworks provides a mechanism to translate international recommendations to a local context and, if building on existing laws, can provide a simpler route to adoption.[3,27] The broader context of LMIC differ from high income countries [28,29] and there may be a need to consider additional legal issues. These include whether the intervention can be implemented effectively within existing legal infrastructure and how the intervention may interplay with production and investment.[30] There may also be international and regional trade and investment law implications where fiscal measures favour local producers, hinder imports or impact foreign investment [18,24,31]. For these reasons, choosing a structure which accords with a country’s existing legal framework and takes account of the multiplicity of legal dimensions is as important as ensuring the efficacy and public health benefit of an intervention.

When looking at how law might inform NCD prevention interventions, a detailed consideration of the legal feasibility of an intervention could inform how an intervention can be formulated to; a) fit within the domestic legal system, b) meet government obligations, c) inform how other legislation and regulations can be harmonised with the intervention, and d) be robust enough to ameliorate the risk of legal challenge.

The methodologies we reviewed included some of these considerations but did not do so exhaustively and were not structured in a manner that allowed the methodology to be used in other legal systems, particularly in the LMIC context. The lack of structure and uniformity in how a comprehensive legal feasibility assessment may be conducted is a key difficulty for assessing legal feasibility in the context of health, and specifically NCD prevention. Though such an assessment can play a large role in shaping the form of an intervention [27], the legality of the intervention is more often than not viewed as a hurdle to be crossed or a binary consideration of whether interventions conflict with domestic law.[1,5] Given the potential value of a thorough legal analysis, there is a need to formulate a comprehensive and structured framework to assess the legal feasibility of interventions for NCD prevention in LMIC.

To facilitate more detailed consideration of these aspects across different legal systems, we have developed a new framework – titled the FELIP framework – which expands the utility of legal feasibility, allows for a multiplicity of legal considerations to be analysed, and can be used in different legal systems. The specific components of the FELIP framework (Figure 1) are i) the potential **F**ormulations, ii) the **E**xisting legal system, iii) **L**aws related to impacted sectors, iv) legal **I**nfrastructure, and v) the **P**rocess. This framework can be used to structure consideration of the legal implications of an intervention and inform whether an intervention should be adopted as well as how it should be formulated. This framework differs from other methodologies in that it considers legal barriers as well as how the law might facilitate and inform the content of an effective health intervention across different legal systems. We utilised this framework to assess the feasibility of an SSB tax in Sub-Saharan African countries but we believe it can be applied to legal interventions for public health more broadly. Each component of our proposed framework is outlined in detail below.

#### Potential formulations of the intervention

The starting point is to delineate the different forms of the intervention being considered. Depending on the intervention and policy-making context, this could include a systematic or scoping review of potential interventions or different formulations of the same type of intervention. This gives the framework a level of flexibility as to what the starting point is. For some interventions, there may be a clear mechanism to be used as is the case with SSB taxation where it is clear that a taxation mechanism must be used. However, for more novel interventions there may be a need to consider a broader range of potential formulations and / or mechanisms aimed at addressing a particular risk factor such as reducing consumption of processed foods.[2] In applying this to our study and SSB taxes, we selected the formulations adopted in Mexico, the United Kingdom and South Africa.

#### Existing legal framework

Once an intervention and its potential formulation(s) have been decided, the existing legal framework should be reviewed to locate both facilitators and barriers to the adoption of the intervention. This includes considerations of governmental authority, national constitutions, existing laws related to the intervention as discussed above as well as existing legal mechanisms which could be adapted for the intervention such as repurposing existing earmarked funds. This is arguably the most critical dimension of the analysis as it allows for the intervention to be located within the existing legal system and attempts to glean potential facilitators for the adoption. If an existing law can be used to adopt the intervention, it would be important to consider how to amend or concretise the intervention to fit within that law. This analysis might also inform whether amendments to existing laws are needed to ensure that the intervention is effective. For assessing an SSB tax, we considered whether there was a need to remove VAT exemptions of sugar and SSBs and tax allowances for SSB manufacturers. Since VAT exemptions have the effect of reducing the price of certain goods, imposing an SSB tax of VAT exempt goods might undermine the efficacy of the intervention.

#### Laws related to impacted sectors

Given the particular legal challenges that arise when addressing CDOH, as discussed above, the legal analysis must go further than just the immediately applicable laws. To ensure the robustness of the intervention as well as potential barriers that can undermine the intervention, a comprehensive legal analysis should consider laws which relate to sectors beyond the specific laws used to adopt the intervention. This will vary considerably depending on the context and intervention but will likely include consideration of trade-related laws at both a domestic and international level. In the case of an SSB tax to reduce sugar consumption, laws relating to trade and agriculture may be implicated depending on whether a country produces its own sugar or imports soft drinks. In LMIC where foreign investment and agriculture can be major contributors to the economy, these sectors may have a significant impact on what kind of interventions are adopted.[9,28] Against this backdrop, changes should be made to the structure and formulation of the intervention to ensure it does not conflict with other laws and does not violate these obligations. Beyond changing the intervention, this analysis can also be used to establish where laws should be amended to harmonise different legislation and ensure the efficacy of interventions.

#### Necessary legal infrastructure

In LMICs, where regulatory and enforcement infrastructure may be underfunded or weak,[9] it is critical to consider whether the legal infrastructure needed to adopt or implement the intervention is in place. This will also be variable but, as discussed above, is related to the legal mechanisms needed for the intervention to be implemented. For a SSB-tax, this may include considerations like monitoring imports and exports, or where it is based on sugar content, regulations allowing the government to monitor nutritional content of beverages. Where this infrastructure is not available, it may be necessary to consider the feasibility of amend the existing infrastructure or to change the formulation of the intervention to a mechanism that does not require this infrastructure.

#### Processes for law-making

The final aspect to consider is the process for adopting the legal intervention. Certain kinds of laws require buy-in from specific policy-makers. Often taxation laws require substantial involvement of the minister or department of finance while laws involving issues of trade may need to emanate from a policymaker such as the ministry of trade. Considering alternative laws and their accompanying processes may be a feasible alternative where there is a lack of buy-in from a key policy maker. Where law-making power is concentrated, this can inform where lobbying efforts should be targeted and give a clear sense of whether an intervention is feasible

#### Utility of the FELIP Framework

Taken together, the different dimensions of the FELIP framework allow us to understand the broad legal landscape, at an international and domestic level in a comprehensive but targeted way. Each of the components of the FELIP framework would enable a policymaker or government legal team to adjust how an intervention will be adopted in a way that takes advantage of opportunities the existing legal system offers while identifying potential barriers to both adoption and effectiveness of the intervention. This more comprehensive approach also facilitates the identification of potential conflicts between laws and provides guidance on where amendments or new laws are needed to implement a particular intervention. This allows for the creation of a legally feasible intervention and adjustments that account for barriers to adoption rather than having to dismiss a potential intervention that conflicts with the existing legal system.

While the approach is comprehensive, it is also focussed and introduces consideration of related sectors that may have an impact on the viability of the intervention without the need to evaluate every existing law and regulation. This approach works particularly well for NCD-related interventions which frequently sit outside the healthcare system and impact on multiple sectors.[9] In addition, it accounts for one of the major challenges NCD prevention efforts often face, challenges arising from international trade agreements.[18] When adapting the framework to local contexts, it may be necessary to include an additional component of pre-emption which may be of relevance in federalist systems – or to adapt the rights approach where a country only has civil political rights or socio-economic rights that are not justiciable.

Despite the value added by an analysis of legal considerations, it is of limited value if it is not supplemented with an analysis of the political, cultural and administrative landscape.[27] Such legal analysis can only assist in combating some of the technical difficulties that arise in developing legal interventions but cannot speak to issues of political will and implementation. In situations where there is political will and sufficient evidence to support the adoption of a legal intervention to improve public health, this legal framework can guide how the intervention can be developed and adopted within the existing legal system.
